# A non-invasive risk score including skin autofluorescence predicts diabetes risk in the general population

**DOI:** 10.1038/s41598-022-26313-9

**Published:** 2022-12-16

**Authors:** Henderikus E. Boersma, Melanie M. van der Klauw, Andries J. Smit, Bruce H. R. Wolffenbuttel

**Affiliations:** 1 Department of Endocrinology, University of Groningen, University Medical Center Groningen, HPC AA31, P.O. Box 30001, 9700 RB Groningen, The Netherlands; 2 Department of Internal Medicine, University of Groningen, University Medical Center Groningen, Groningen, The Netherlands

**Keywords:** Endocrine system and metabolic diseases, Biomarkers

## Abstract

Increased skin autofluorescence (SAF) predicts the development of diabetes-related complications and cardiovascular disease. We assessed the performance of a simple model which includes SAF to identify individuals at high risk for undiagnosed and incident type 2 diabetes, in 58,377 participants in the Lifelines Cohort Study without known diabetes. Newly-diagnosed diabetes was defined as fasting blood glucose ≥ 7.0 mmol/l and/or HbA_1c_ ≥ 6.5% (≥ 48 mmol/mol) or self-reported diabetes at follow-up. We constructed predictive models based on age, body mass index (BMI), SAF, and parental history of diabetes, and compared to results with the concise FINDRISC model. At 2nd visit to Lifelines, 1113 (1.9%) participants were identified with undiagnosed diabetes and 1033 (1.8%) participants developed diabetes during follow-up. A model comprising age, BMI and SAF yielded an AUC of 0.783 and was non-inferior to the concise FINDRISC model, which had an AUC of 0.797 to predict new diabetes. At a score of 5.8, sensitivity was 78% and specificity of 66%. Model 2 which also incorporated parental diabetes history, had an AUC of 0.792, and a sensitivity of 74% and specificity of 70% at a score of 6.5. Net reclassification index (NRI) did not improve significantly (NRI 1.43% (− 0.50–3.37 p = 0.15). The combination of an easy to perform SAF measurement with age and BMI is a good alternative screening tool suitable for medical and non-medical settings. Parental history of diabetes did not significantly improve model performance in this homogeneous cohort.

## Introduction

The worldwide prevalence of diabetes mellitus was estimated in 2019 to be just under half a billion people. This number is expected to rise to 578 million by 2030 and 700 million by 2045. More than 90% of these have type 2 diabetes (T2D) and approximately 50% of individuals with T2D are unaware of their disease. The economic impact of diabetes care is significant^1^ and estimated to rise with the projected increased prevalence^2^. Prevention, as well as early detection and treatment of T2D are essential to reduce the incidence of complications. Screening programs result in T2D being diagnosed 3.3 years earlier on average^3^. Although randomized clinical trials show no beneficial effect on a population level^4,5^, there seems to be a reduction in healthcare costs, cardiovascular disease (CVD), and mortality for those found to have diabetes by screening^6^. Furthermore, screening can select people at high risk of developing diabetes. Multiple trials have demonstrated lifestyle modification's effectiveness in preventing or delaying the onset T2D in high-risk groups. This effectiveness is especially pronounced in individuals with higher FINDRISC scores^7^. The strategy of using a diabetes risk score with subsequent lifestyle intervention in high-risk groups is likely to be cost-effective^8^.

The formation of advanced glycation endproducts (AGEs) plays an important role in the pathophysiology of micro- and macrovascular complications in diabetes. Skin Autofluorescence (SAF) can assess the accumulation of AGEs in the skin^9^. SAF is elevated in people with T2D compared with age-matched controls^10^. Furthermore, SAF is already elevated in people with metabolic syndrome and undiagnosed diabetes in cross-sectional studies^11^. Moreover, in a prospective study, elevated SAF was significantly associated with new-onset diabetes after correction for confounders^12^. Measuring SAF could be a replacement for invasive and clinical impractical variables, like waist circumference, in existing screening tools. As suggested before, SAF might be a viable screening tool used in G.P. practices and pharmacies. Since patients are more willing to participate in screening if they were approached while visiting healthcare for other reasons^13^, the easy, fast and non-invasive nature of SAF measurement could reach patients not otherwise motivated to evaluate their risk of developing diabetes.

The aim of this study was to construct fast and straightforward risk models based on SAF measurement and demographic variables to identify individuals at high risk for undiagnosed DM2 and incident DM2, suitable for medical and non-medical settings, and compare these models with the well-known FINDRISC model.

## Methods

### Participants

Subjects included were participants from the Lifelines Cohort Study^14^. Lifelines is a large prospective multi-generational population-based study examining the interaction between genetic and environmental factors in the development of chronic diseases and healthy aging. Residents living in the northern region of the Netherlands aged 25–50 years and their family members were invited to participate. Baseline data collection has been completed between 2006–2013 for more than 167,000 participants. Follow-up visits are scheduled every 4–5 years and are ongoing. All individuals provided written informed consent before participating in the study, which was approved by the Medical Ethics Review Committee of the University Medical Center Groningen.

**Figure 1:**
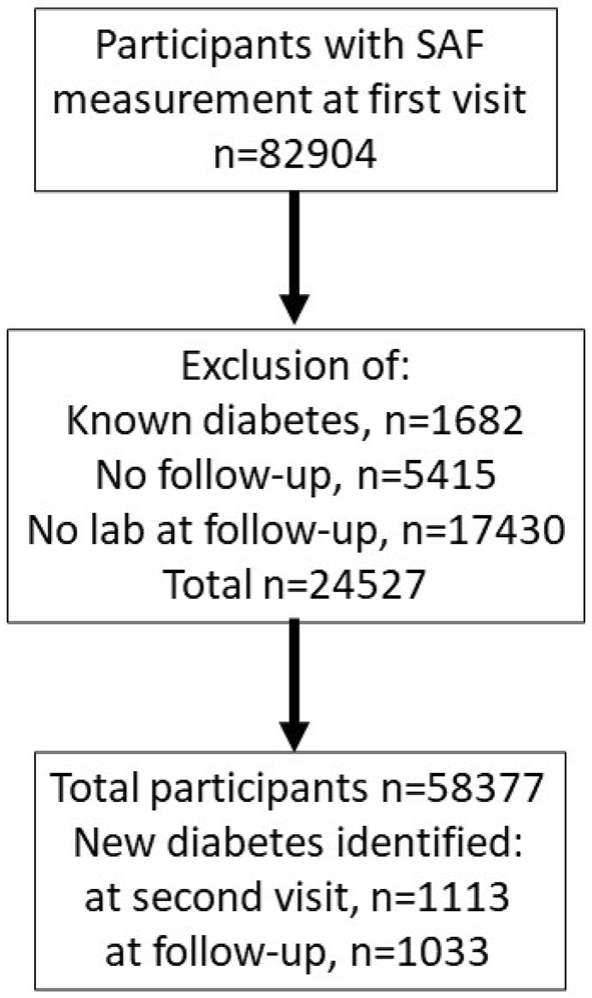
Disposition of lifelines participants.

For the current analysis, we evaluated 82,904 participants of whom validated SAF measurement were available at baseline. There were no relevant differences between those with and without SAF measurements in gender distribution, age, and glycaemic variables. We excluded participants with known self-reported type 1 or type 2 diabetes (n = 1682), those without documented follow-up (n = 5415), and those in whom no follow-up measurements of fasting glucose or HbA_1c_ were available (n = 17,430). Also, we excluded participants with extreme SAF values (< 0.8AU or > 4.5AU, n = 22), as such values likely represent measurement failures (for example, by incomplete skin coverage from external light). This resulted in 58,377 individuals available for analysis (Fig. 1).

### Clinical examination

During the first visit to the Lifelines screening center, information on medical history, health status, and smoking habits was collected using self-administered questionnaires. Smoking status was classified into never, former or current smoking. The use of medication was verified using the Anatomical Therapeutic Chemical Classification System by a research assistant. Weight (rounded to the nearest 0.1 kg), height and waist circumference (rounded to the nearest 0.5 cm) were measured while participants were wearing no shoes and light clothing. Body mass index (BMI) was calculated as kg/m^2^. Blood pressure and heart rate were measured with an automated Dinamap monitor (GE Healthcare, Freiburg, Germany), for a total of 10 measurements every 10 min. Values of blood pressure (BP) and heart rate were registered by averaging the final three readings. Skin autofluorescence (SAF) was measured at the volar side of the forearm using an AGE Reader (Diagnoptics Technologies, Groningen, the Netherlands) as described previously^9^. SAF is expressed in arbitrary units (AU), while taking skin color into account. Reproducibility of repeated SAF measurements, including measurements taken over a single day and seasonal variance, showed a relative error of 5%^15^.

### Biochemical measurements

During a second visit, which usually was 4–8 weeks later, blood was drawn between 8 and 10 a.m. while participants were in the fasting state. Biochemical measurements were performed on the same day. Glycated haemoglobin (HbA_1c)_ was measured in EDTA-anticoagulated blood on a Cobas Integra 800 CTS analyzer (Roche Diagnostics, Almere, The Netherlands) with a National Glycohemoglobin Standardized Program certified turbidimetric inhibition immunoassay method. Blood glucose was measured with a hexokinase method. Serum concentrations of creatinine, total cholesterol, HDL-cholesterol, LDL-cholesterol, and triacylglycerol were measured on a Roche Modular P chemistry analyzer (Roche, Basel, Switzerland. Estimated (e) GFR was calculated with the CKD-EPI (Chronic Kidney Disease Epidemiology Collaboration) formula^16^.

### Follow-up

Follow-up questionnaires were administered by mail or online approximately 1.5 and 3 years later, and a new clinic visit was to be performed after 4 years later. These follow-up questionnaires evaluated changes in health status and medication. Also, after 4 years biochemical measurements including fasting blood glucose and HbA1c were repeated.

### Calculations, definitions and statistical analyses

Undiagnosed diabetes was based on a fasting blood glucose ≥ 7.0 mmol/l and/or HbA_1c_ ≥ 48 mmol/mol (≥ 6.5%) measured at the second visit. For incident diabetes at follow-up, the same definition was used as well as self-reported newly-developed T2D. Diagnosis of metabolic syndrome was based on the revised National Cholesterol Education Program criteria from 2004 by the American Heart Association.

The original FINDRISC publication included a full model and a concise model^17^. Variables in the concise FINDRISC model are age, BMI, waist circumference, use of antihypertensive agents and history of high blood glucose. Previously, a simplified variant of this concise FINDRISC model with the addition of SAF was created^18^. For the current analysis, this adjusted model was used as a starting point. Grouping of age and BMI were based on the original FINDRISC score. Receiver operating characteristic (ROC) curve analysis was used to define groups for SAF; cut-off values were chosen based on their 50%, 90% and 97.5% specificity for detecting baseline diabetes. Later studies added family history to the original FINDRISC model^19^; therefore, we reanalyzed the dataset with inclusion of parental history of T2D, defined as the number of parents with T2D.

Multivariable logistic regression analysis was then performed to determine the association between predefined age group, BMI group and classes of SAF and incident diabetes (model 1). For model 2, we added parental diabetes history as a determinant. The point score values were estimated based on the β coefficients of the logistic regression model and are presented for both models. Five risk groups were pre-defined based on the proportion of participants, defined as 0-50th percentile, 51–80th percentile, 81–90th percentile, 91–98th percentile and 99–100th percentile of the combined point score, thus covering the spectrum from very low (group 1) to very high (group 5) risk. As a sensitivity analysis, we repeated these calculations for participants aged 40 and above, and for those with established hypertension.

The discriminatory ability of the different models was estimated using the area under the ROC (AUROC) curve, with R packages PredictABEL^20^, pROC^21^, and ggplot2^22^. We directly compared both models with the net reclassification improvement (NRI) index^18^. Finally, we evaluated the prevalence of important risk factors, namely presence of metabolic syndrome, treatment with antihypertensive or lipid-lowering agents, and smoking habits, for each of the five risk groups.

Data are presented as mean ± standard deviation (SD) when normally distributed. Otherwise, median and interquartile range was used. Means were compared between groups with analysis of variance. The Mann–Whitney *U* test was used when variables were not normally distributed. The χ^2^ test was used to analyze categorical variables. Analyses were conducted with SPSS Statistics (Version 25, IBM, Armonk, NY, USA). P-values < 0.05 were considered statistically significant.

### Ethics approval and consent to participate

This study was approved by the medical ethical review committee of the University Medical Center Groningen. All participants provided written informed consent before participating in the study and all methods were carried out in accordance with relevant guidelines and regulations for human subjects.

## Results

Table 1 provides the clinical characteristics of the study population. The prevalence of undiagnosed diabetes at the second visit was 1113 (1.9%). Diagnosis of new diabetes at follow-up, after a median period of 4 years, was either self-reported (n = 525) or established by elevated glucose (n = 528) and/or elevated HbA_1c_ (n = 294), respectively, for a total of 1033 (1.8%) participants. This resulted in a total of 2146 participants with “newly-detected diabetes”. These participants had higher SAF (1.92 ± 0.42 vs. 2.20 ± 0.48, P < 0.001). They were also significantly older, more likely male, had larger waist circumference and higher BMI, blood glucose, and HbA_1c_. In addition, their blood pressure and total and LDL-cholesterol were higher, with a larger proportion using blood-pressure-lowering drugs and statins (all *P* < 0.01).

**Table 1:** Baseline characteristics of the participants according to follow-up diabetes status.

Characteristic	Control N = 56,231	New diabetes N = 2146	P-value
Sex (*n*; male/female)	23,115/33,116	1155/991	< 0.001
Age (years)	45.0 ± 12.1	53.6 ± 11.8	< 0.001
BMI (kg/m^2^)	25.8 ± 4.0	29.8 ± 5.0	0.009
Waist (cm)	103 ± 13	104 ± 14	0.009
Systolic BP (mmHg)	126 ± 15	135 ± 17	< 0.001
Diastolic BP (mmHg)	74 ± 9	78 ± 10	< 0.001
Heart rate (bpm)	71 ± 11	73 ± 12	< 0.001
Creatinine (µmol/l)	74 ± 13	75 ± 15	< 0.001
eGFR (ml/min/1.73m^2^)	96 ± 15	91 ± 16	0.003
Total cholesterol (mmol/l)	5.1 ± 1.0	5.3 ± 1.1	< 0.001
HDL-cholesterol (mmol/l)	1.49 ± 0.39	1.27 ± 0.37	< 0.001
LDL-cholesterol (mmol/l)	3.2 ± 0.9	3.4 ± 1.0	< 0.001
Triacylglycerol (mmol/l)	1.15 ± 0.73	1.82 ± 1.44	< 0.001
Glucose (mmol/l)	4.9 ± 0.5	6.7 ± 2.0	< 0.001
HbA_1c_ (mmol/mol)	36 ± 3	45 ± 11	< 0.001
HbA_1c_ (%)	5.5 ± 0.3	6.3 ± 1.0	< 0.001
Current smoking (%)	19.1	24.0	< 0.001
Former smoking (%)	33.0	41.2	< 0.001
% using BP-lowering therapy	10.5	34.6	< 0.001
% using statins	4.4	19.9	< 0.001
Skin autofluorescence (AU)	1.92 ± 0.42	2.20 ± 0.48	< 0.001

Data are presented as numbers, percentages, or mean ± SD. *BMI* body mass index, *BP* blood pressure, *eGFR* estimated glomerular filtration rate, *HbA1c* glycated haemoglobin, *HDL* high-density-lipoprotein, *LDL* low-density lipoprotein.

There are small differences between the participants who were undiagnosed at the second visit compared to those with new onset diabetes at follow-up. Those who were undiagnosed at baseline were slightly older (mean age 54.7 vs. 52.4 years), and had slightly higher SAF Z-score (mean + 0.29 vs. + 0.16 AU). As 525 of 1033 participants had self-reported new diabetes at follow-up, they have been diagnosed by their G.P. inbetween baseline and follow-up, and are likely to have started treatment with lifestyle interventions and/or medication, thereby limiting their exposure to hyperglycaemia.

**Figure 2:**
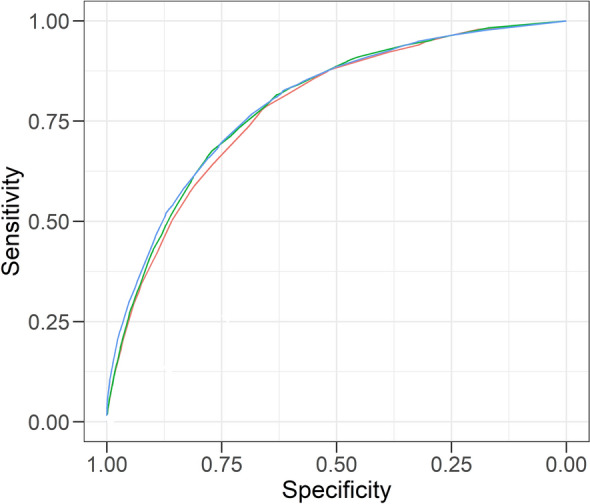
ROC curve of the two simple SAF-based predictive models vs. the concise FINDRISC model. Red line: Model 1, comprising age group, BMI group, SAF group (AUC 0.783, 95% CI 0.774–0.793); Green line: Model 2, comprising age group, BMI group, SAF group and number of parents with diabetes (AUC 0.792, 95% CI 0.783–0.802); Blue line: The concise FINDRISC model (AUC 0.797, 95% CI (0.787–0.806).

Logistic multivariable regression analyses with age, BMI and SAF group showed that all three variables were significant predictors of newly-detected diabetes (Table 2). The ROC curve for model 1 yielded an area under the curve (AUC) of 0.783 (95% CI 0.774–0.793); the addition of number of parents with T2D as a fourth variable in model 2 (Table 2) yielded a similar AUC of 0.792 (95% CI 0.783–0.802). In comparison (Fig. 2), the concise FINDRISC model yielded an AUC of 0.797 (95% CI 0.787–0.806). There was also no difference in AUC between both SAF-based models and FINDRISC when new diabetes at second visit (AUC varying from 0.790 to 0.797) and new diabetes during follow-up (AUC varying from 0.758 to 0.771) were assessed separately.

**Table 2:** Regression coefficients (point scores) obtained in the two multivariable logistic regression models.

		Model 1	Model 2
Age group (years)	18–44	1	1
	45–55	1.822	1.748
	56–64	2.640	2.607
	≥ 65	3.593	3.747
BMI group (kg/m^2^)	< 25.0	1	1
	25.0–29.9	2.792	2.725
	≥ 30.0	8.693	8.254
SAF group (AU)	< 1.90	1	1
	1.90–2.49	1.615	1.594
	2.50–2.89	2.091	2.092
	≥ 2.90	3.264	3.261
n of parents with diabetes	0		1
	1		1.781
	2		2.546

*AU* arbitrary units, *BMI* body mass index, *SAF* skin autofluorescence.

The prevalence of diabetes rose markedly as the model score increased. For model 1, at a score of 5.8, the sum of sensitivity and specificity was at a maximum; this resulted in a sensitivity of 78% and a specificity of 66%; for model 2 this cut-off value was 6.5 and had a sensitivity of 74% and specificity of 70%. For the concise FINDRISC model, the sum of sensitivity and specificity was at a maximum at ≥ 7, which resulted in a sensitivity of 76% and specificity of 69%.

As intended, 20% (model 1 n = 11,550, model 2 n = 11987) of participants were classified as medium risk or higher (Table 3). At medium risk or higher, 1200 cases (56% (95% CI 53.8–58)) with new diabetes were detected for model 1 and 1252 cases (58% (95% CI 56.2–60.4)) for model 2 (sensitivity). Of those without diabetes, 45881 (82% (95% CI 81.3–81.9)) for model 1 and 45,496 (81% (95% CI 80.6–81.2)) for model 2 were classified as low or very low risk (specificity). The probability of new diabetes at medium or higher risk was 10.4% (95% CI 10.0–10.8) for model 1 and 10.4% (95% CI 10.1–10.8) for model 2 (positive predictive value). Participants with low and very low risk had a probability of 98.0% (95% CI 97.9–98.1) to be without diabetes during follow-up (negative predictive value).

**Table 3:** Incidence of diabetes according to risk group classification.

Risk group	Model 1	Model 2
Group 1: very low	0.9 (264/29,323)	0.9 (266/29,187)
Group 2: low	3.9 (682/17,504)	3.7 (628/17,203)
Group 3: medium	7.2 (389/5438)	7.1 (429/6005)
Group 4: high	11.3 (573(/5050)	12.1 (613/5050)
Group 5: very high	22.4 (238/1062)	22.5 (210/932)

Data represented as % of positive cases (number of positive cases/total number of cases) Model 1 comprises age group, BMI group, SAF group; model 2 comprises age group, BMI group, SAF group and number of parents with diabetes.

As part of our sensitivity analyses, incidences of diabetes in participants aged ≥ 40 years and in those with hypertension are presented in Additional Fig. 1A and 1B. As expected, percentage of newly-detected diabetes was higher in both analyses, with similar results for both SAF-based models. Although low eGFR may increase AGE accumulation and SAF values, a post-hoc analysis revealed no interaction between SAF and eGFR for diabetes prediction. Based on the current 4-year follow-up, we estimated 10-yr incidence of diabetes to vary from 1.6% (very low risk) to 40% (very high risk, Additional Fig. 2).

Table 4 shows the number and percentage of participants classified into five risk categories who were reclassified into other categories by adding number of parents with T2D to the model. This proportion was lowest in the very low-risk group and highest in the very high-risk group. Reclassification was only to an adjacent risk group. In total, 21% of participants were reclassified. As shown by observed diabetes risk, most of the reclassification into higher-risk groups is accurate. Net reclassification did not improve significantly by adding number of parents with T2D (NRI 1.4% (95% CI 0.5–3.4 p = 0.15)).

**Table 4:** Reclassification between risk classification according to Model 1 and Model 2.

Predicted diabetes risk (model 1)	Predicted risk of diabetes (model 2)						
	1	2	3	4	5	Total	Reclassified
Group 1	25,803	3520				29,323	3520 (12%)
	0.8%	1.9%					
Group 2	3384	12,980	1140			17,504	4524 (26%)
	2.0%	3.9%	9.3%				
Group 3		703	4092	643		5438	1346 (25%)
		7.7%	6.6%	10.3%			
Group 4			773	3886	391	5050	1164 (23%)
			7.0%	11.6%	17.6%		
Group 5				521	541	1062	521 (49%)
				18.6%	26.1%		
Total	29,187	17,203	6005	5050	932	58,377	12,334

Cardiovascular risk factors were more prevalent in higher-risk groups (Table 5). With each higher risk group, mean BMI increased, from 23.7 to 33.0 kg/m^2^. The prevalence of metabolic syndrome rises from 4.5% at very low risk to 56.2% at very high risk. Similarly, use of blood-pressure-lowering drugs and statin usage is more frequent in higher-risk groups. In contrast, the lower-risk group contains more current smokers, while the higher-risk groups contain more former smokers.

**Table 5:** Association between risk classification according to Model 2 and existing cardiovascular risk factors.

Risk group	n	BMI (kg/m^2^)	Metabolic syndrome (%)	Treated w. BP-lowering (%)	Treated w. statin (%)	Current smoking (%)	Former smoking (%)
Very low	29,187	23.7	4.6	4.1	1.4	21.0	25.5
Low	17,203	26.0	15.3	13.7	6.8	18.4	40.2
Medium	6005	29.8	31.5	21.9	10.9	16.2	40.8
High	5050	33.2	49.9	26.4	9.5	16.9	41.9
Very high	932	33.0	57.4	46.5	19.8	14.2	53.9

Diagnosis of metabolic syndrome is based on the revised National Cholesterol Education Program criteria from 2004 by the American Heart Association (19). Diagnosis is established if at least three out of five criteria are met: (1) SBP ≥ 130 mmHg and/or DBP ≥ 85 mmHg and/or use of antihypertensive medication; (2) HDL-cholesterol < 1.03 mmol/l in men and < 1.30 mmol/l in women and/or use of HDL-cholesterol-elevating medication; (3) triglyceride levels > 1.70 mmol/l and/or use of triglyceride-lowering medication; (4) waist circumference ≥ 102 cm in men and ≥ 88 cm in women; (5) fasting glucose levels > 5.6 mmol/l.

## Discussion

In the current study, we showed that a simplified model comprising age class, BMI class and number of parents with diabetes combined with a SAF measurement has similar performance for diabetes detection compared with the concise FINDRISC model, at initial screening and prospectively during 4 years follow-up.

Previously the study by Waateringe et al.^12^ showed the value of SAF in predicting incident diabetes, which to date is the only study on the predictive value of SAF on incident diabetes. Several earlier cross-sectional studies on the value of SAF to detect prevalent diabetes have been published. SAF was superior in detecting OGTT-defined impaired glucose tolerance versus fasting glucose and HbA_1c_^23^. A different study showed that a decision model consisting of SAF, BMI and family history for diabetes was superior to fasting blood glucose and non-inferior to HbA_1c_ and FINDRISC in detecting undiagnosed diabetes and impaired glucose tolerance^24^. As mentioned before, the study by Fokkens et al.^18^ showed that SAF improved FINDRISC’s model performance for the detection of undiagnosed diabetes, and our simplified model including SAF, age and BMI performed similar compared to FINDRISC.

In the current study, the discrimination for FINDRISC was somewhat lower (AUC 0.797 vs. 0.857) compared to the original publication, as risk models often perform better for the population they were designed for. After recalibration, the simplified model performed non-inferior for the outcome of undiagnosed prevalent diabetes and incident diabetes after four years of follow-up compared to the concise FINDRISC model. Thus, SAF measurement can replace FINDRISC model variables while preserving performance. This results in an alternative screening tool for settings where collecting adequate medical history and medication use would be infeasible. Although the AGE reader requires a one-time investment, it should be noted that the concise FINDRISC model requires the availability of at least an earlier (invasive) blood glucose measurement.

Given the high risk of complications due to diabetes, a higher sensitivity is desirable. However, this would result in a more significant proportion of the population to be referred for additional laboratory testing. Additionally, the effect of diabetes screening and subsequent early treatment on a population level has not yet been shown to reduce mortality or cardiovascular disease^4^. However, lifestyle intervention in the prevention of diabetes was especially effective in higher-risk individuals^7^. Therefore, we chose multiple risk categories with an increased risk of developing diabetes. We propose a single glucose measurement for persons with medium risk and up, and subsequent 3-yearly glucose measurements only for the high and very high-risk group. At the same time, we encourage a lifestyle intervention for all people with medium risk and up. The awareness of increased risk for the development of diabetes has been found to be a good motivator and predictor of a successful lifestyle intervention^25^.

The addition of number of parents to the risk score resulted in a minor improvement, AUC 0.792 vs. 0.783 (Fig. 2). Although many participants were reclassified**,** only 3% (1843) were reclassified at intermediate risk. Reclassification did not significantly improve prediction, NRI 1.4% (95% CI 0.5–3.4 p = 0.15). The vast majority of Lifelines participants have a Caucasian ethnicity (98%) and are born in the Netherlands (97%). This homogeneity could explain the limited additional value of family history of diabetes to the modeling. The addition of family history to the original FINDRISC model was based on the DETECT-2 cohort, a combination of multiple cohorts with participants from Europe, Australia and Africa^19^. Additional validation in a heterogeneous population is needed to confirm the additional value of number of parents to the risk score.

Another striking finding is the lower prevalence of current smokers and higher prevalence of former smokers in the higher risk groups. This strongly suggests earlier successful smoking cessation as a consequence of risk factor evaluation and modification by general practitioners. It is encouraging to see more successful smoking cessation in these high-risk groups. However, smoking cessation has also been associated with a higher short-term risk of weight increase and developing diabetes^26^.

In individuals with diabetes SAF is elevated by increased formation of AGEs. However, as we show in the current study, SAF is already elevated before the development of diabetes. In part this can be explained by the association of SAF with the components of the metabolic syndrome, known risk factors for the development of diabetes^11^. However, after correcting for these factors SAF remains an independent predictor of diabetes. Crossectional studies show an association between dietary AGEs intake and insulin resistance^27^. In animal studies exogenous AGEs are linked to reduced peripheral insulin responsiveness and involved in islet β-cell damage in both T1D and T2D^28^. However, the effect of dietary AGEs on insulin resistance in healthy volunteers is conflicting in small diet intervention trials^29,30^.

## Limitations

One limitation of the current study is the availability of only a single measurement of fasting plasma glucose and HbA_1c_. Both the WHO and ADA advise a repeated measurement in asymptomatic people. Second, fasting glucose and HbA_1c_ measurements at follow-up were available in only 77% of participants. As 50% of incident diabetes was diagnosed by laboratory testing, some participants with diabetes have been missed. We corrected for this by excluding all participants with missing glucose and HbA_1c_ measurements at follow-up. Furthermore, of the participants with self-reported diabetes at follow-up, a proportion did not have elevated plasma glucose or HbA_1c_ at laboratory screening. It is unknown whether this is due to the prescription of blood-glucose-lowering medication. Third, data on physical activity, vegetables and fruit consumption in Lifelines were not compatible with the full FINDRISC model; therefore, only the concise FINDRISC model could be used. Also, a history of high blood glucose was not addressed as such in the Lifelines questionnaire; participants with this history were only identified when they filled a free space in the questionnaire regarding diabetes. The impact of this was probably limited as in prior validation studies of FINDRISC in the Dutch population, only 0.7–1.6% of participants reported such a history^31^. We refrained from assessing calibration of different models, as it should be noted that the original FINDRISC publication used drug-treated diabetes as the outcome. Finally, information regarding family history of diabetes was incomplete; therefore, we could not compare the performance with the updated FINDRISC model, as an alternative number of parents with T2D was used.

## Conclusions

The combination of an easy to perform SAF measurement with age and BMI is a good alternative screening tool suitable for medical and non-medical settings. Furthermore, family history of diabetes did not improve model performance in this homogeneous cohort.

## Supplementary Information

Supplementary Figures.

## Data Availability

The manuscript is based on data from the Lifelines Cohort Study. Lifelines adheres to standards for data availability, and allows access for reproducibility of the study results. The data catalogue of Lifelines is publicly accessible at www.lifelines.nl. The dataset supporting the conclusions of this article is available through the Lifelines organisation (e-mail: research@lifelines.nl). For data access, a fee is required.

## Abbreviations

T2D: Type 2 diabetes
CVD: Cardiovascular disease
AGEs: Advanced glycation endproducts
SAF: Skin autofluorescence
BMI: Body mass index
BP: Blood pressure
AU: Arbitrary units
SD: Standard deviation
HbA_1c_: Glycated haemoglobin
AUC: Area under the curve
NRI: Net reclassification index
