# Supplementary material for: A non-invasive risk score including skin autofluorescence predicts diabetes risk in the general population
Source: Sci Rep. 2022 Dec 16;12:21794. doi: 10.1038/s41598-022-26313-9 (PMC9758123; doi:10.1038/s41598-022-26313-9)
Supplement: Supplementary file 1 — Supplementary Figures. [file 41598_2022_26313_MOESM1_ESM.docx]

**Supplementary Information**

**Additional figure 1a. Incidence of diabetes according to risk classification in participants of age 40 years and above**

Model 1 comprises age group, BMI group, SAF group; model 2 comprises age group, BMI group, SAF group and number of parents with diabetes.

**Additional figure 1b. Incidence of diabetes according to risk classification in participants with hypertension (according to the Metabolic Syndrome criteria, >130/80 or treated)**

Model 1 comprises age group, BMI group, SAF group; model 2 comprises age group, BMI group, SAF group and number of parents with diabetes.

**Additional Figure 2. Extrapolated 10-year Incidence of diabetes according to risk classification**

Model 1 comprises age group, BMI group, SAF group; model 2 comprises age group, BMI group, SAF group and number of parents with diabetes. Incidence calculated as new cases at baseline screening together with 2.5 times the number of cases detected in the first 4 years of follow-up
